# Magnetic resonance imaging of the cranial nerves in infectious, neoplastic, and demyelinating diseases, as well as other inflammatory diseases: a pictorial essay

**DOI:** 10.1590/0100-3984.2021.0042

**Published:** 2022

**Authors:** Mariana Dalaqua, Felipe Barjud Pereira do Nascimento, Larissa Kaori Miura, Marcio Ricardo Taveira Garcia, Alcino Alves Barbosa Junior, Fabiano Reis

**Affiliations:** Hôpitaux Universitaires de Genève, Service de Radiologie, Genève, Switzerland.; Imaging Department, Hospital Israelita Albert Einstein, São Paulo, SP, Brazil.; Department of Radiology, Universidade Estadual de Campinas (Unicamp), Campinas, SP, Brazil.; Diagnósticos da América S/A (DASA), Barueri, SP, Brazil.

**Keywords:** Cranial nerves/physiopathology, Cranial nerve diseases/diagnostic imaging, Demyelinating diseases/diagnostic imaging, Magnetic resonance imaging/methods, Nervos cranianos/fisiopatologia, Doenças dos nervos cranianos/diagnóstico por imagem, Doenças desmielinizantes/ diagnóstico por imagem, Ressonância magnética/métodos

## Abstract

The cranial nerves, which represent extensions of the functional structures of the brain, traverse the head and neck. They are connected to various cranial structures and are associated with several diseases. An in-depth understanding of their complex anatomy and normal imaging appearance allows the examiner to identify and characterize abnormalities with greater precision. One important tool for evaluating the cranial nerves is contrast-enhanced magnetic resonance imaging, especially that employing three-dimensional steady-state free precession sequences, which provide high soft-tissue and spatial resolution, despite the slen-derness of the nerves. In most cases of cranial nerve abnormalities, the imaging findings are nonspecific. Therefore, to narrow the differential diagnosis, it is necessary to take a full patient history, perform a focused physical examination, and order laboratory tests. In this pictorial essay, we review, illustrate, and discuss, from a pathophysiological perspective, infectious, neoplastic, and demyelinating disorders, as well as other inflammatory disorders, affecting the cranial nerves, the aim being to provide a practical, tangible reference for radiologists to use in daily practice.

## Abstract (pt)

Os nervos cranianos, que representam extensões das estruturas funcionais do cérebro, atravessam a cabeça e o pescoço. Eles estão conectados a várias estruturas cranianas e estão associados a várias doenças. Uma compreensão profunda de sua complexa anatomia e aparência normal por imagem permite ao examinador identificar e caracterizar as anormalidades com maior precisão. Uma ferramenta importante para avaliar os nervos cranianos é a ressonância magnética com contraste, especialmente as sequências tridimensionais *steady-state free precession*, que proporcionam alta resolução espacial e de partes moles, apesar da fina espessura dos nervos. Na maioria dos casos, os achados radiológicos não são específicos. Para estreitar o diagnóstico diferencial é necessário fazer uma anamnese completa do paciente, realizar um exame físico dirigido e solicitar testes laboratoriais. Neste ensaio iconográfico revisamos, ilustramos e discutimos, sob uma perspectiva fisiopatológica, os distúrbios infecciosos, neoplásicos, inflamatórios e desmielinizantes, visando a ser uma referência prática e tangível para a prática diária dos radiologistas.

## INTRODUCTION

Infections of the central nervous system (CNS) are common, arising from hematogenous dissemination, contiguous propagation (usually from the paranasal sinuses, temporal bone, or skull base), traumatic injuries, or surgical manipulation. Those most frequently affected are the optic, trochlear, trigeminal, abducens, and facial nerves (cranial nerves II, I V, V, VI, and VII, respectively). On magnetic resonance imaging (MRI), abnormal leptomeningeal enhancement along cerebral surfaces and the cisternal segments of cranial nerves is a hallmark of infection and should be correlated with the results of cerebrospinal fluid (CSF) analysis.

Neoplastic conditions of the cranial nerves may be primary or secondary, and the former may be further divided into benign and malignant conditions. To make the diagnosis, it is useful to determine the location of abnormal enhancement and its character (regular or nodular); to identify cystic, necrotic, or hemorrhagic components; and to correlate those data with other imaging findings in the CNS.

Inflammatory and demyelinating diseases may arise from parainfectious and autoimmune processes, presenting with a monophasic, relapsing–remitting, or progressive course. Some of these diseases may cause cranial nerve thickening and abnormal enhancement. The correlation with specific local alterations in the brainstem and spinal cord help distinguish specific etiologies.

In a previous article^(1)^, we reviewed congenital, traumatic, and vascular diseases of the cranial nerves. This pictorial essay goes further, illustrating infectious, neo-plastic, and demyelinating diseases, as well as other inflammatory diseases. Although all of the pathologic conditions we review in this paper share the common finding of abnormal contrast enhancement in cranial nerves, each has specific epidemiological, clinical, biochemical, and imaging findings.

### INFECTIOUS DISEASES

#### Neuroborreliosis

**Figure 1:**
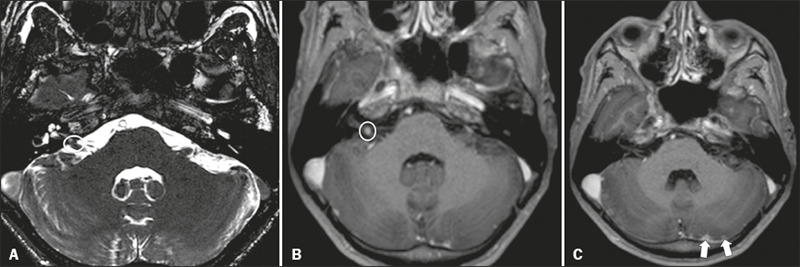
Neuroborreliosis. 50-year-old male presenting with unilateral facial palsy. **A:** Fast imaging employing steady-state acquisition MRI sequence showing a nodular lesion with low signal intensity (white circle) next to the cisternal segments of the right cranial nerves VII and VIII. B: Gadolinium-enhanced T1WI showing uniform contrast enhancement (white circle) of a nodular lesion at the right internal auditory canal. There is also thickening and enhancement of right abducens nerve (cranial nerve VI) between the pons and the pyramidal tract. **C:** Gadolinium-enhanced T1WI showing areas of nodular enhancement (arrows) in the left cerebellar hemisphere.

Neuroborreliosis is a systemic inflammatory disease, with a worldwide distribution, that occurs after a bite from the deer tick *Ixodes scapularis* and inoculation of complex spirochetes of the genus *Borrelia* (*B. burgdorferi, B. afzelii*, or *B. garinii*). The disease occurs in stages, with a variety of signs and symptoms. When there is neurologic involvement, which occurs in 10–15% of infected individuals, it is known as Lyme neuroborreliosis, the mechanisms of which include vasculitis, cytotoxicity, neurotoxic mediators, and immune reactions. Lymphocytic meningitis, cranial neuropathy (particularly facial nerve palsy), and radiculoneuritis constitute the classic triad of acute Lyme neuroborreliosis. An MRI scan can depict enhancement of multiple cranial nerves, with or without signal changes in the white matter (Figure 1). In endemic areas, Lyme neuroborreliosis should be considered when a patient presents with unilateral or bilateral cranial nerve palsy, especially when that is preceded by erythema migrans^(2)^.

#### Racemose neurocysticercosis

**Figure 2:**
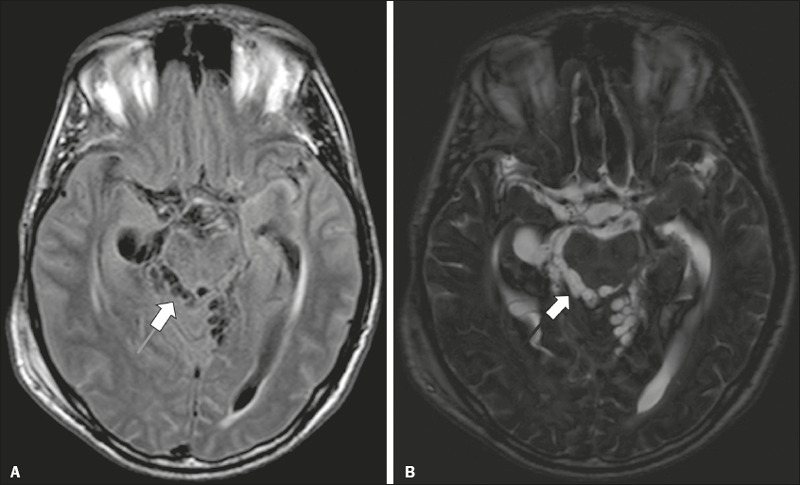
Racemose neurocysticercosis. 23-year-old male presenting with mental confusion, hallucinations, and right trochlear nerve (cranial nerve IV) palsy. **A:** FLAIR MRI sequence showing cysts in the subarachnoid space of the posterior fossa. **B:** Fast imaging employing steady-state acquisition MRI sequence showing confluent cysts in the region of the right trochlear nerve pathway (arrow), consistent with the symptoms.

Cysticercosis is a parasitic infection resulting from ingestion of eggs from an adult *Taenia solium* pork tapeworm, and its neurologic findings may include cranial nerve pathology^(3)^. A cysticercus may have one of two shapes: cystic (vesicle containing a scolex, designated *Cysticercus cellulosae*) or grape-like (a cluster of vesicles without a scolex, designated *Cysticercus racemosus*). Racemose cysts, which are typically located in the subarachnoid space, cisterns, or ventricles, can grow to large dimensions, occasionally displacing or constricting adjacent structures (Figure 2). The consequences of neurocysticercosis include vasculitis caused by an inflammatory response to antigens in the parenchyma, basal leptomeninges, compression/displacement of cranial nerves, and ventricular enlargement. The diagnosis is based on the results of a CSF analysis and radiological examinations. Fluid-attenuated inversion recovery (FLAIR) sequences, diffusion-weighted imaging (DWI), and three-dimensional constructive interference in steady-state sequences facilitate visualization of the scolex in *C. cellulosae* cysts, allowing them to be distinguished from those of *C. racemosus*.

#### Tuberculous meningitis

**Figure 3:**
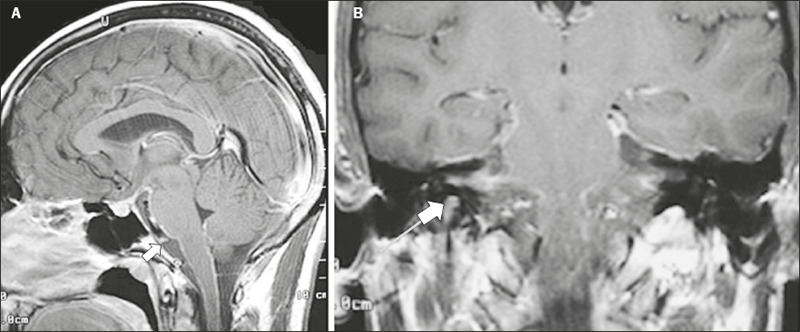
Tuberculous meningitis. 30-year-old male presenting with progressive headache, seizures, and reduced visual acuity. Analysis of the CSF showed increased adenosine deaminase, suggesting tuberculous meningitis. Gadolinium-enhanced sagittal and axial T1WI showing diffuse leptomeningeal enhancement along the surface of brainstem (arrow in **A**), together with enhancement of the facial nerve (cranial nerve VII, arrow in **B**) and the vestibulocochlear nerve (cranial nerve VIII) in the right cerebellopontine angle.

Tuberculosis is a disease that is caused by infection with *Mycobacterium tuberculosis* and has a worldwide distribution. Neurotuberculosis represents neurologic involvement secondary to hematogenous dissemination and may present as exudative leptomeningitis or as paren-chymatous lesions (tuberculoma, abscess, or cerebritis). Cranial neuropathy, which may be caused by vasculitis or inflammation, is seen in 17–70% of patients with CNS tuberculosis^(4)^. Radiological features of CNS tuberculosis include basal leptomeningeal enhancement, hydrocephalus, and infarctions in the supratentorial brain parenchyma or brainstem, as well as, in some cases, cranial nerve enhancement (Figure 3).

#### Neurosyphilis

**Figure 4:**
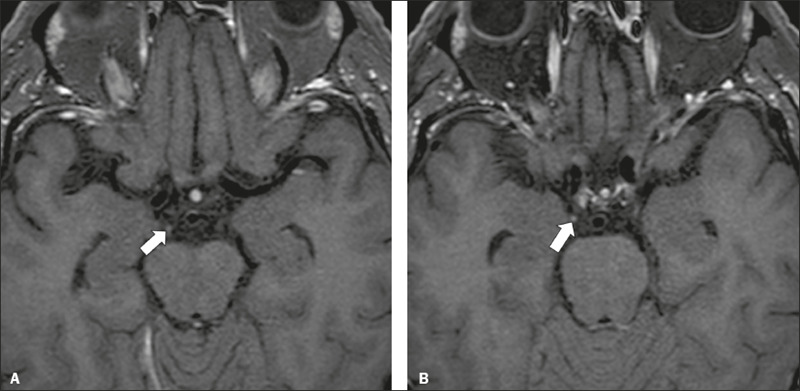
Neurosyphilis. Non–HIV-infected 67-year-old male presenting with cranial nerve and vascular involvement detected on HR-VWI. Arrows indicate enhancement of the right oculomotor nerve (cranial nerve III).

Neurosyphilis is a reemerging infectious disease that can be transmitted sexually and vertically. It has a wide spectrum of neurological manifestations and can mimic many other infectious and inflammatory diseases, as well as tumors and demyelinating processes. Recently, highresolution vessel wall imaging (HR-VWI) has proven useful to differentiate ischemic lesions from vessel wall inflammation associated with infectious vasculitis, and HR-VWI sequences can depict cranial nerve enhancement in infectious conditions, as demonstrated by Feitoza et al.(^5^,^6^) and illustrated in Figure 4.

#### Ramsay Hunt syndrome

Ramsay Hunt syndrome is one of several causes of peripheral facial nerve palsy in the geniculate ganglion. It occurs due to reactivation of latent infection with the varicella-zoster virus, which causes inflammation and neural degeneration^(7)^. Facial nerve palsy results from edema and compression of the facial nerve (cranial nerve VII) within the narrow facial canal and is accompanied by sensorineural hearing loss, tinnitus, vertigo, nystagmus, and erythematous vesicular rash around the ear (herpes zoster oticus). The MRI features of facial nerve palsy are nonspecific, although contrast enhancement at the fundus of the inner ear canal, representing inflammatory changes, is common. In rare cases, Ramsay Hunt syndrome occurs as a component of multiple cranial nerve involvement, especially that of the trigeminal, glossopharyngeal, and vagus nerves (cranial nerves V, IX, and X, respectively). Edema and enhancement of trigeminal brainstem nuclei can also be seen.

#### Bell’s palsy

The condition known as Bell’s palsy is a type of facial nerve palsy that can be idiopathic or of suspected viral etiology.

It is the most common cause of acute spontaneous peripheral facial paralysis. Herpes simplex virus activation is the likely cause of Bell’s palsy in most cases, although there is no well-established, widely available method of confirming a viral mechanism in clinical practice. Inflammation starts at the geniculate ganglion, extending proximally and distally, causing facial palsy due to neural edema inside the narrow facial canal^(7)^. Laboratory tests are usually not indicated. However, because at least 10% of affected patients are diabetic, a blood glucose test may be performed. There are several differential diagnoses, including Lyme disease, Ramsay Hunt syndrome, neoplasia, and sarcoidosis. A detailed history-taking usually facilitates the diagnosis. The MRI findings are nonspecific and typically include unilateral facial nerve enhancement.

#### Paracoccidioidomycosis

Paracoccidioidomycosis is a fungal disease caused by *Paracoccidioides* spp. (*P. brasiliensis* or *P. lutzii*), which can result in systemic granulomatous infection. It is most commonly observed in adult male laborers in rural subtropical areas of Latin America^(8)^. Involvement of the CNS occurs in 10% of chronic cases, and patients with CNS paracoccidioidomycosis may present with focal neurological deficits, cognitive changes, weight loss, headache, and seizures. The most common CNS lesions are due to granulomatous reactions in the supratentorial and infratentorial parenchyma. However, in some cases, there can be leptomeningeal and pachymeningeal inflammation caused by cranial nerve inflammation. The diagnosis of CNS paracoccidioidomycosis is established by direct detection of the fungus, through biopsy or resection of a granuloma, CSF analysis, or culture. Although the MRI findings are nonspecific, they can contribute to the diagnosis by depicting ring-enhancing lesions in the parenchyma. Leptomeningeal or pachymeningeal enhancement may also be seen.

#### Echinococcosis

**Figure 5:**
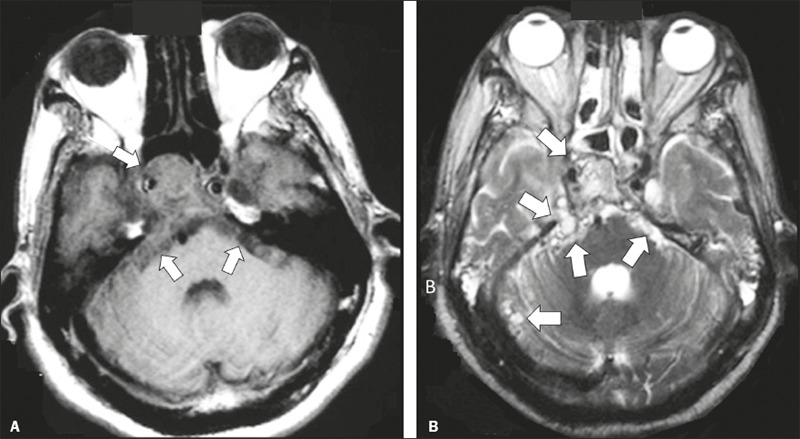
Echinococcosis. 45-year-old male presenting with a 4-month history of cognitive impairment, preceded by headaches, nausea, fever, and confusion. On physical examination, bilateral impaired extrinsic ocular movements, anisocoria, and meningism were observed. Axial T1WI (**A**) and axial T2WI (**B**). Arrows indicate cysts in the cerebellopontine cisterns and in the right juxtasellar region. The abducens, facial, and vestibulocochlear nerve (cranial nerves VI, VII, and VIII, respectively) were affected. Autopsy revealed multiple hydatid cysts corresponding to the MRI findings.

Echinococcosis is a zoonotic infection caused by adult or larval stages of the tapeworms *Echinococcus multilocularis* and *Echinococcus granulosus*, which cause alveolar and cystic echinococcosis, respectively. Alveolar echinococcosis is uncommon and occurs secondary to intrahepatic growth of parasitic larvae. Cestodes reach other organs (especially the lungs, brain, and bones) by direct infiltration or hematogenous spread. In individuals with alveolar echinococcosis, cranial nerve palsies have been reported and the symptoms depend on the location of the lesions^(9)^. Wild canids and domestic dogs act as the definitive hosts of *E. granulosus*, the causative agent of cystic echinococcosis (also known as hydatid disease), and human infection with *E. granulosus* occurs through contact with contaminated animals. Neurological involvement, which is rare, may include the brain parenchyma or subarachnoid space (Figure 5).

### NEOPLASTIC DISEASES

#### Petrous apex meningioma

Meningioma is the most common extra-axial tumor and is also the most common non-glial primary CNS tumor.

Among petrous apex meningiomas, the most common types are petroclival and cerebellopontine angle meningiomas. Petroclival meningiomas arise from the medial aspect of the petrous apex and progress through its wall or to Dorello’s canal, whereas cerebellopontine angle meningiomas arise from the posterior petrous apex and extend to the internal auditory canal. The MRI findings are variable, although dural-based masses with intense contrast enhancement, often hypointense to brain tissue on T1-weighted imaging (T1WI) and isointense on T2WI, are common findings^(10)^.

#### Optic nerve pilocytic astrocytoma

Pilocytic astrocytoma is the most common primary brain tumor in children. The structures most often affected are the cerebellum, optic nerve/chiasm, hypothalamus, and thalami. It is commonly associated with neurofibromatosis type 1, especially when it occurs in the optic pathways. In individuals with optic nerve pilocytic astrocytoma, MRI shows well-circumscribed, slow-growing tumors that are hypointense or isointense to brain tissue on T1WI and hyperintense on T2WI/FLAIR sequences, with little or no edema. Enhancement is variable, usually intense. Another common presentation is a cystic lesion with a mural nodule^(11)^.

#### Schwannoma

**Figure 6:**
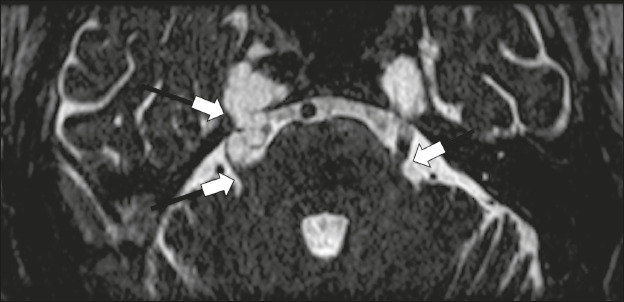
Trigeminal cystic schwannoma. An 84-year-old asymptomatic male in whom the lesion was discovered as an incidental finding. Fast imaging employing steady-state acquisition MRI sequence showing an extra-axial, predominantly cystic expansive mass along the cisternal segment of right cranial nerve V (arrows on the left), extending anteriorly to Meckel’s cave. The arrow on the right indicates the normal trigeminal nerve.

Schwannoma is a benign nerve sheath tumor derived from Schwann cells. It is the most common tumor of the peripheral nervous system. The cranial nerve that is most often affected (in 90% of cases) is the vestibulocochlear nerve (cranial nerve VIII), followed by the trigeminal nerve (cranial nerve V). Acoustic schwannomas grow into the cerebellopontine angle, displacing the brainstem and cerebellum. In most cases, these originate from within the internal auditory canal, the dilation of which is an early radiological sign of tumor growth. Bilateral acoustic schwannomas are pathognomonic of neurofibromatosis type 2, which is a phakomatosis that also increases the chances of meningiomas and ependymomas (multiple inherited schwannomas, meningiomas, and ependymomas), as illustrated in Figure 6.

#### Esthesioneuroblastoma

**Figure 7:**
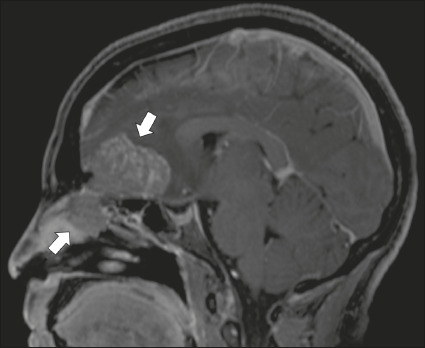
Esthesioneuroblastoma. Gadolinium-enhanced sagittal T1WI showing a heterogeneously enhancing mass traversing the cribriform lamina, with intracranial and extracranial components (arrows). Corresponding DWI, T2WI, and FLAIR sequence (not presented) showing low apparent diffusion coefficients in solid components, right olfactory bulb involvement, and vasogenic edema in the adjacent right frontal lobe.

An esthesioneuroblastoma, also known as an olfactory neuroblastoma, is a malignant neuroectodermal tumor arising from the olfactory mucosa in the upper nasal cavity. It typically affects men, being most common among those in their twenties, with a second peak among those in their sixties. It usually involves the cribriform plate, extending to the upper nasal cavity. Smaller esthesioneuroblastomas will show local spread in the nose and sinuses, whereas larger ones may invade anterior the cranial fossa, brain, and dura mater. Imaging depicts a dumbbell-shaped mass with its narrow segment at the level of cribriform plate, causing local bone destruction^(12)^, as demonstrated in Figure 7. The Kadish staging system classifies esthesioneuroblastomas as follows: stage A— limited to the nasal cavity; stage B—limited to the nasal cavity and paranasal sinuses; and stage C—extending beyond the nasal cavity and paranasal sinuses, including the skull base, intracranial compartment, and orbit, as well as distant metastases.

#### Temporal bone paragangliomas

**Figure 8:**
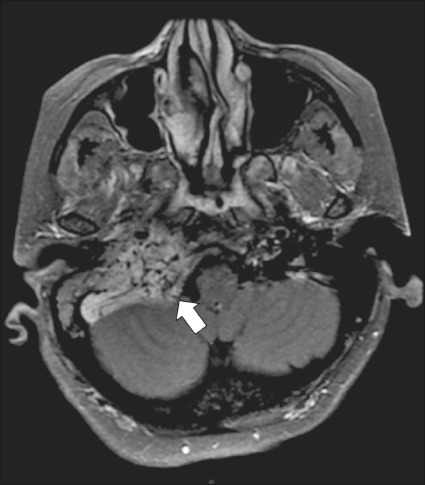
Jugulotympanic paraganglioma. 61-year-old female presenting with hearing loss, right facial palsy, dysphagia, and Horner syndrome. Gadolinium-enhanced axial spin-echo T1WI showing an enhancing lesion (arrow) centered in right jugular foramen, involving the glossopharyngeal, vagus, and accessory nerves (cranial nerves IX, X, and XI, respectively). The lesion extended superiorly to the internal auditory canal—involving the facial and vestibulocochlear nerves (cranial nerves VII and VIII, respectively—and the middle ear, extending inferiorly to the hypoglossal nerve (cranial nerve XII) and carotid canals (not shown).

Temporal bone paragangliomas, also known as jugulotympanic paragangliomas, are tumors that arise from the extra-adrenal neuroendocrine system, are composed of neural crest progenitor cells, and are usually benign. Involvement of the jugular foramen is most prevalent among individuals in their fifties or sixties, representing the main cause of pulsatile tinnitus associated with a vascular retrotympanic mass, as identified on physical examination^(13)^ and depicted in Figure 8

#### Carotid body paraganglioma

Paragangliomas may also occur in the carotid space, presenting as a mass between the internal and external carotid arteries. Cranial neuropathy, which is seen in 20% of patients with carotid body paraganglioma, can involve the glossopharyngeal, vagus, accessory, and hypoglossal nerves (cranial nerves IX, X, XI, and XII, respectively). Unenhanced MRI shows punctate vascular flow voids (“salt and pepper” pattern), whereas contrast-enhanced MRI shows a mass with rapid, intense contrast enhancement^(13)^.

### DEMYELINATING DISEASES

#### Multiple sclerosis

Multiple sclerosis is a multifactorial disorder with an inflammatory autoimmune component, characterized by inflammatory and immune attacks on oligodendrocytes, resulting in demyelination and axonal injury. It is the most common disabling CNS disease in young adults, more often affecting women than men and being most prevalent among individuals 20–40 years of age. The lesions are predominantly located in the white matter, typically in the periventricular region (Dawson’s fingers), corpus callosum, subcortical regions, brainstem, and spinal cord, although they may occur in the gray matter as well. Isolated cranial nerve involvement is uncommon, and cranial nerve injury is frequently seen in conjunction with other brain lesions. The optic and trigeminal nerves (cranial nerves II and V, respectively) are the most affected, although cases of isolated oculomotor nerve (cranial nerve III) dysfunction have also been reported^(14)^. The general imaging features of acute multiple sclerosis are swollen, enhancing areas that are isointense on T1WI and usually bright on T2WI/FLAIR sequences, with or without restricted diffusion on DWI. In the chronic phase, the lesions are usually hypointense and nonenhancing on T1WI (“black holes”), being bright on T2WI/FLAIR sequences, sometimes with visible volume loss or cavitation. Cranial nerves may appear enlarged with marked enhancement^(15)^.

#### Neuromyelitis optica spectrum disorders

Neuromyelitis optica spectrum disorders constitute a group of autoimmune demyelinating CNS diseases that preferentially affect the optic nerve, periventricular regions (including the area postrema), and spinal cord. They are typically associated with an immune response (neuromyelitis optica-immunoglobulin G antibody against aquaporin 4 channels). In general, the imaging features are longitudinally extensive T2WI hyperintensity within the spinal cord (spanning three or more vertebral segments), as well as enlargement and enhancement of the optic nerve (cranial nerve II). On T2WI, the abnormality tends to involve the spinal cord more centrally or the entire cross-section of the cord, unlike the more peripheral involvement seen in multiple sclerosis^(16)^.

#### Chronic inflammatory demyelinating polyneuropathy

**Figure 9:**
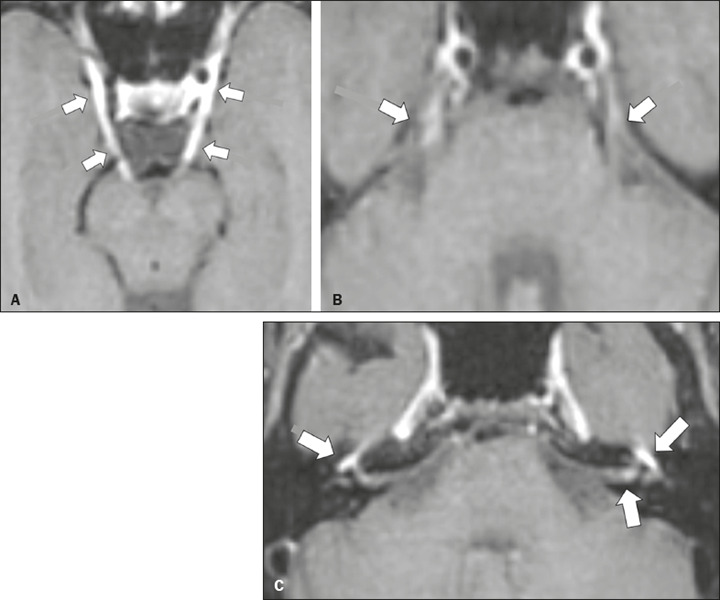
Chronic inflammatory demyelinating polyneuropathy. 40-year-old female presenting with chronic polyneuropathy with new diffuse motor exacerbation. Gadolinium-enhanced three-dimensional sampling perfection with application-optimized contrasts using different flip angle evolution black-blood T1WI showing bilateral thickening and enhancement of cranial nerves III (A, oculomotor nerve), V (B, trigeminal nerve), VI (abducens nerve, not shown), and VII (C, facial nerve). Contrast enhancement was also seen in multiple cervical rootlets, in the brachial plexus, and in the cauda equina nerve roots (not shown).

Chronic inflammatory demyelinating polyneuropathy is an acquired sensorimotor idiopathic neuropathy that primarily involves the distal motor nerve. Cranial nerve involvement is seen in 5% of patients, in which MRI may show thickened, enhancing nerves (Figure 9). It has a relapsing-remitting or progressive course of demyelination and may respond to immunosuppressive therapy. The classic symptoms are symmetric proximal and distal weakness, together with sensory loss in two or more limbs. Hyporeflexia or areflexia may also be seen^(17)^.

### OTHER DISEASES

#### Trigeminal neuritis

**Figure 10:**
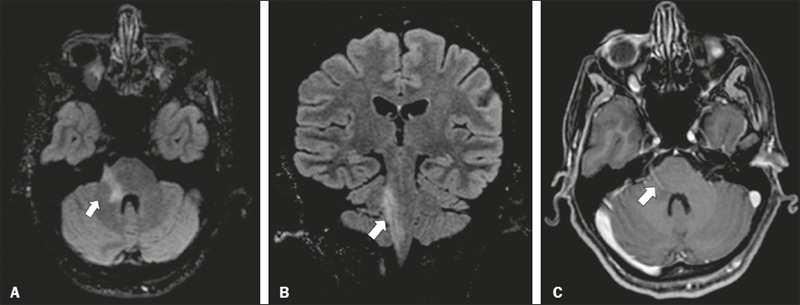
Trigeminal neuritis. Right facial pain and palsy. Axial and coronal FLAIR sequences (**A** and **B**, respectively) showing high signal intensity in the right brainstem (arrows), involving the superior and middle cerebellar peduncles, together with the lateral aspect of the pons, posterior aspect of the bulb, region of the trigeminal nuclei, and pontine fibers of the right trigeminal nerve, as well as the intra-axial pathways of the right facial nerve and its nervus intermedius. **C:** Gadolinium-enhanced axial T1WI showing enhancement of the pontine fiber pathways of the trigeminal nerve (arrow).

In most cases, trigeminal neuritis is caused by an inflammatory reaction to herpes simplex virus type 1, which lies dormant within the trigeminal ganglion. Retrograde spread along the preganglionic segment of the trigeminal nerve (cranial nerve V) into the brainstem, albeit rare, may cause rhombencephalitis^(18)^, which may involve other cranial nerves as well. The MRI findings include high signal intensity of the inflamed brainstem segment on T2WI and contrast enhancement of the affected nerve on T1WI (Figure 10). We present images of involvement of the nucleus of the facial nerve (cranial nerve VII) ipsilateral to the trigeminal neuritis.

#### Non-Langerhans cell histiocytosis

Hemophagocytic syndrome, Erdheim-Chester disease, and Rosai-Dorfman disease are all forms of non-Langerhans cell histiocytosis, which is a rare multisystemic disease of the monocyte-macrophage system, with variable severity and radiological presentation. Among children under 15 years of age, it has an incidence of 0.2–2.0 cases per 100,000 population, with a slight predominance of males. The cranial and intracranial changes seen on MRI may include craniofacial bone and skull base lesions, with or without soft-tissue involvement, as well as intracranial and extra-axial changes in the hypothalamic-pituitary region, leptomeninges, cranial nerves, and circumventricular organs^(19)^.

## CONCLUSION

This pictorial essay could help narrow the differential diagnosis of nonspecific imaging findings of cranial nerves, which typically include thickening and enhancement. Although infectious, neoplastic, demyelinating, and inflammatory diseases of the cranial nerves may look alike on MRI, scrutiny of other brain imaging findings, correlated with the clinical and biochemical data, facilitates the differential diagnosis. Therefore, radiologists play a key role in making the accurate diagnosis of such diseases.
